# Association between Quantitative Classification of Renal Surface Nodularity and Early Renal Injury in Patients with Arterial Hypertension

**DOI:** 10.1155/2022/1553700

**Published:** 2022-03-04

**Authors:** Jun Zhou, Jiule Ding, Jie Chen, Qiong Wu, Dehui Xiang, Wei Xing

**Affiliations:** ^1^Department of Radiology, The Third Affiliated Hospital of Soochow University, Changzhou 213003, China; ^2^School of Electronics and Information Engineering, Soochow University, Suzhou, Jiangsu 216006, China

## Abstract

**Background:**

This study sought to explore the association between quantitative classification of renal surface nodularity (qRSN) based on computed tomography (CT) imaging and early renal injury (ERI) in patients with arterial hypertension.

**Methods:**

A total of 143 patients with a history of hypertension were retrospectively enrolled; clinical information (age, sex, hypertension grade, and hypertension course), laboratory tests, and qRSN were collected or assessed. The subjects were divided into an ERI group (*n* = 60) or a control group (CP, *n* = 83) according to ERI diagnosis based on the following criteria: cystatin C > 1.02 mg/L. Univariate analysis and multiple logistic regression were used to assess the association between ERI and qRSN. A receiver operating characteristic curve (ROC) was performed to compare multiple logistic regression models with or without qRSN for differentiating the ERI group from the control group.

**Results:**

In univariate analysis, hypertension grade, hypertension course, triglycerides (TG), and qRSN were related to ERI in patients with arterial hypertension (all *P* < 0.1), with strong interrater agreement of qRSN. Multiple logistic regression analysis showed an area under the ROC curve of 0.697 in the model without qRSN and 0.790 in the model with qRSN, which was significantly different (*Z* = 2.314, *P*=0.021).

**Conclusion:**

CT imaging-based qRSN was associated with ERI in patients with arterial hypertension and may be an imaging biomarker of early renal injury.

## 1. Introduction

Hypertension is a condition characterized by systemic persistent arterial hypertension, affecting approximately 874 million adults worldwide [1–3]. Hypertension can damage multiple organs throughout the body and is particularly closely related to renal injury [2, 4–8]. Renal injury worsens arterial hypertension, and elevated blood pressure increases the risk of renal injury, forming a vicious circle [1, 9]. In general, early diagnosis of renal injury is extremely important for the effective treatment and prognosis of patients with arterial hypertension. However, some patients may not seek treatment until renal injury is in advanced stages, as early renal injury (ERI) is asymptomatic.

Hypertension can cause renal cortical fibrosis, renal tubule atrophy, and preferential loss of irregular superficial nephrons because of variable arteriosclerosis of feeding blood vessels [10, 11]. The abovementioned pathological changes can manifest as a typical imaging finding of renal surface nodularity [10], one of the imaging biomarkers of ERI in patients with arterial hypertension, which has been confirmed at the pathological level but not from a clinical practice view. Therefore, the aim of this study was to explore the relationship between quantitative classification of renal surface nodularity (qRSN) and ERI in patients with arterial hypertension. We present the following article in accordance with the STROB reporting checklist.

## 2. Methods

### 2.1. Clinical Characteristics

**Figure 1:**
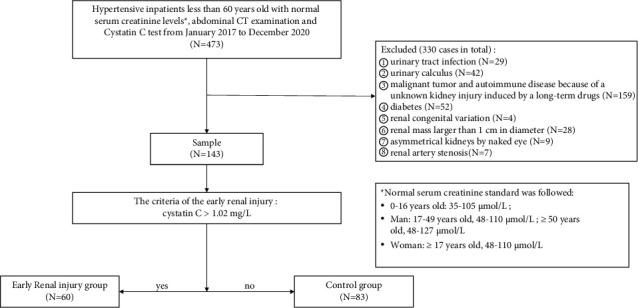
Flow chart of the selection of patients with arterial hypertension.

This study retrospectively included inpatients with hypertension aged 18–60 years admitted to a local hospital from January 2017 to December 2020. All patients underwent an enhanced abdominal CT scan and laboratory tests during their hospitalization. All patients had normal serum creatinine (Figure 1). Patients with a history of urinary tract infection (*n* = 29), urinary calculus (*n* = 42), malignant tumor, or autoimmune disease because of an unknown kidney injury induced by long-term drugs (*n* = 159), diabetes (*n* = 52), and renal congenital variations such as lobulated kidney, ectopic kidney, abnormal renal rotation, polycystic kidney (*n* = 4), renal masses or cysts >1 cm in diameter (*n* = 28), asymmetrical kidneys (*n* = 9), or renal artery stenosis (*n* = 7) were excluded (Figure 1). Clinical data collected included age, sex, and hypertension grade on admission and hypertension course. Laboratory tests on admission included cystatin C, serum creatinine, total cholesterol (TC), triglycerides (TG), and low-density lipoprotein (LDL) laboratory results (Annex 1). Hypertension and hypertension grade were defined according to the 2018 edition of the Chinese hypertension guidelines [12]. Hypertension course was divided into three categories: 0–9 years, 10–19 years, and greater than or equal to 20 years.

Cystatin C is typically used as an indicator of early renal injury in clinical situations [13, 14]. Therefore, the patients were divided into an ERI group or a control group (CP) according to the ERI criteria: cystatin C > 1.02 mg/L.

### 2.2. Imaging Acquisition

Several CT types of equipment (GE Optima 64, Toshiba Aquilion One 320, Siemens Sensation 16, and Somatom Definition Flash) were used at a single medical center. The CT scan parameters were as follows: tube voltage of 120 kV, tube current of 10 mA–370 mA, slice thickness of 5 mm, slice increment of 5 mm, a field of view of 35 cm^2^∼40 cm^2^, and matrix 512 × 512. The CT scan of the corticomedullary phase was conducted at 30–35 s after starting iopromide contrast (Ultravist 350 or 370, Bayer Schering Pharma, Berlin, Germany) injection at an injection flow rate of 2.5–5.0 mL/s and a dose of 1.0–1.5 mL/kg.

## 3. Kidney Segmentation and Quantitation of Renal Surface Nodularity

**Figure 2:**
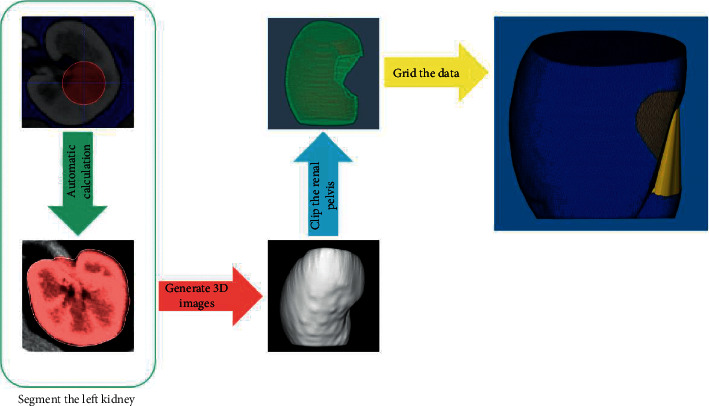
In threshold mode (ITK SNAP), the lower threshold and upper threshold were 20 and 300, respectively, and then an aspherical bubble was placed within the renal parenchyma on CT images. The regional competition force and smoothing force were 0.900 and 0.300, respectively, to automatically segment the left kidney (red area). Three layers of images (15 mm in thickness) at the upper and lower poles of the left kidney were then removed. 3D images of the kidney were generated. Afterwards, the renal pelvis was clipped. Finally, the data were gridded [15].

**Figure 3:**
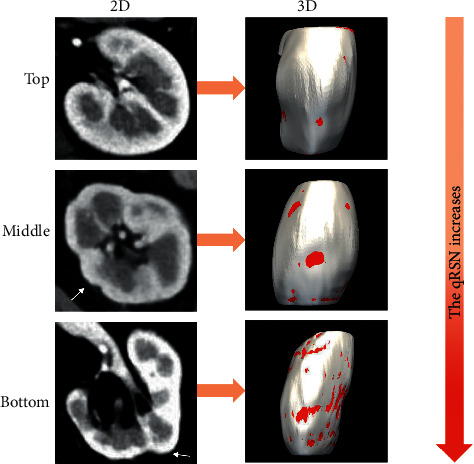
Axial and 3D diagrams of qRSN on the renal surface of patients with arterial hypertension (white arrows and red areas). **Top** (the left kidney of a 44-year-old man in the control group), his qRSN is 1.428. **Middle** (the left kidney of a 56-year-old woman in the ERI group), renal surface nodularity (RSN) was detected on the axial CT image and 3D diagram. Her qRSN is 1.610. **Bottom** (left kidney of a 42-year-old man in ERI group), RSN was detected on the axial CT image and 3D diagram. His qRSN is 2.155.

CT image data at the corticomedullary phase (DICOM files) were analyzed by a radiologist (Wang KX). Twenty of 143 cases were sampled randomly and analyzed by another radiologist (Wang T) to assess interrater agreement of qRSN. The automated algorithm in ITK SNAP (version 3.8, https://www.itksnap.org) was employed to segment the kidney, as shown in Figure 2. A 3D surface mesh of the segmented kidney was generated as indicated in a previous study [15], and point coordinates were adjusted by using a windowed sinc function interpolation kernel [16]. The Euclidean distance between the generated 3D and the smoothed 3D surface mesh was computed. The median Euclidean distance was used to quantify qRSN and was normalized to the minimum value (Figure 3).

### 3.1. Statistical Analysis

All analyses were performed using SPSS or MedCalc. Categorical variables are expressed as frequencies (%). Continuous variables are expressed as the mean ± standard deviation. The Bland–Altman test was performed to assess interrater agreement of qRSN. The linear relationship between age and qRSN was tested by Pearson's correlation. Variables between the ERI and CP groups were compared using *χ*^2^, Wilcoxon rank sum, or two independent sample *t* tests, as appropriate. To avoid eliminating potentially meaningful variables, *P* < 0.1 was considered statistically significant. Multiple logistic regression analysis was applied to explore the relationship of qRSN with ERI. *P* < 0.05 was considered statistically significant. A variance inflation factor value <10 was considered to indicate no obvious collinearity.

## 4. Results

### 4.1. Clinical Data and Laboratory Tests

There were 60 cases in the ERI group and 83 in the CP group. Patients in the ERI group were 48.100 ± 9.152 years old, while those in the CP group were 48.060 ± 8.069 years old. There were 78.3% (47/60) males in the ERI group and 69.9% (58/83) in the CP group. There was no significant difference in age, sex, TC, or LDL between the ERI and CP groups (*P* > 0.1) (Table 1). However, there were more cases of hypertension grade 3 in the ERI than in the CP group (26.7% vs. 2.4%), more cases with hypertension course ≥20 years in the ERI group (16.7% vs. 4.8%), and higher TG in the ERI group (2.031 ± 1.022 mmol/L vs. 2.005 ± 1.745 mmol/L). Therefore, hypertension grade, hypertension course, and TG were included in the multiple logistic regression analysis.

### 4.2. Quantitative Classification of Renal Surface Nodularity

**Figure 4:**
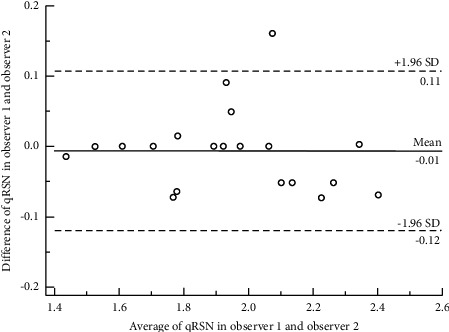
The Bland–Altman diagram of quantitative classification of renal surface nodularity (qRSN). Interrater agreement of qRSN was strong because most of the small circles with interrater differences of qRSN were in the area of 95% limits of agreement. Twenty cases were selected randomly from the full cohort and analyzed for interrater agreement in this study.

**Figure 5:**
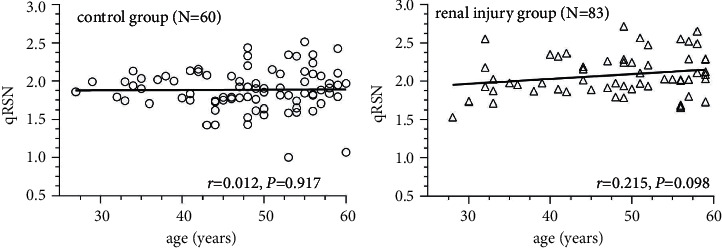
Scatter plots of quantitative classification of renal surface nodularity (qRSN) among patients of different ages. qRSN did not correlate significantly with age in either the control (*r* = 0.012, *P*=0.917) group or the early renal injury group (*r* = 0.215, *P*=0.098).

The Bland–Altman plot (Figure 4) depicts the strong interrater agreement of qRSN. There was no difference in qRSN between the two observers (1.941 ± 0.263 vs. 1.947 ± 0.270, *t* = −0.076, *P*=0.940) (Table S1). In addition, qRSN did not correlate with age in either the ERI or CP groups (*r* < 0.3) (Figure 5).

qRSN was significantly higher in the ERI group than in the CP group (2.080 ± 0.271 vs. 1.885 ± 0.270, *Z* = −3.862, *P* < 0.001) (Table 1). Therefore, it was included in the multiple logistic regression analysis.

### 4.3. Multiple Logistic Regression Analysis

**Figure 6:**
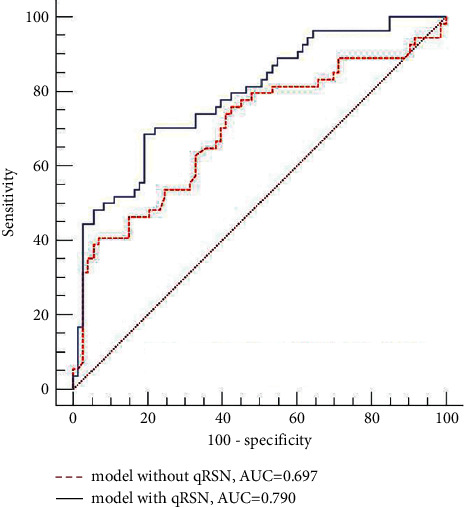
Comparison of receiver characteristic curves of models with and without qRSN for differentiating the renal injury group from the control group. The area under the receiver operating characteristic curve in the logistic regression model combined with qRSN was larger than that in the model without qRSN (*Z* = 2.314, *P*=0.021).

No collinearity in hypertension grade, hypertension course, TG, and qRSN was detected (all variance inflation factor value <10). Multiple logistic regression analysis (Table 2) showed that qRSN correlated independently with ERI (OR = 14.365, 95% confidence interval: 2.746∼75.146, *P*=0.002). The area under the ROC curve was 0.697 in the model without qRSN and 0.790 in the model with qRSN, which was significantly different (*Z* = 2.314, *P*=0.021) (Figure 6).

## 5. Discussion

Renal injury in patients with arterial hypertension has always been a focus of clinical attention. Detection of ERI is very important for patients with arterial hypertension. Previous studies have found that renal surface nodularity may play a role in improving the evaluation and staging of chronic kidney disease [17]. On this basis, this study further quantified the classification of renal surface nodularity and analyzed the relationship between qRSN and ERI for the first time. Cystatin C, a sensitive serum marker of preclinical nephropathy, is not affected by muscle conditions and is more sensitive than creatinine [13, 14, 18, 19]. Therefore, this study used cystatin C to define ERI in patients with arterial hypertension and establish a relationship between qRSN and ERI. Importantly, this study found that qRSN was an independent index for ERI, and the OR value was 14.365.

Denic et al. conducted studies with respect to renal injury risk factors, renal microscopic results, and renal macroscopic results and found that high blood pressure can cause both nephron hypertrophy and nephron atrophy [10]. Glomerulosclerosis and tubular atrophy caused by ischemic degeneration initially occur in superficial nephrons [20, 21], causing superficial nephrons to atrophy and local fibrosis in the renal cortex. At the same time, a residual healthy glomerulus exhibits compensatory hypertrophy and hyperfiltration. Atrophy of the cortical nephron and secondary glomerular hypertrophy around atrophied cortical nephrons appear as nodular changes on the renal surface. Considering the compensatory effect of normal nephrons, renal function in patients with RSN may not decrease significantly until more than 50% renal nephron injury occurs.

Overall, renal injury and blood pressure affect each other through bone mineral metabolism, the renin-angiotensin-aldosterone system, and other mechanisms, forming a vicious cycle [16–19]. The long-term vicious cycle of hypertension and renal injury can aggravate the development of the abovementioned pathological changes and the formation of nodules on the surface of the kidney, and the process of nodule formation on the renal surface reflects the long-term dynamic influence of hypertension on the kidney. Hence, qRSN may be a multidimensional indicator. In this study, the logistic regression model combined with qRSN had greater power than that without qRSN for assessing ERI in patients with arterial hypertension.

Although the number of renal surface nodules increases with age [17], there was no significant correlation between age and qRSN in this study, which may be related to different selection criteria. Previous research conclusions were based on comparisons of an older group (64–75 years old) with a younger group (18–25 years old). In our study, patients over 60 years old were not included because they have a higher likelihood of nonhypertension-related renal injury.

This study had several limitations. First, the images derived from several CT types of equipment and thicker layers may reduce the accuracy of qRSN, but as it was detected in both groups, it did not affect the conclusion but rather improved the generalizability of the research conclusions. Second, renal biopsy is the gold standard for diagnosing ERI, but it is an invasive method and not included in this study. Third, this was a single-center retrospective study, and the selective admission of patients may have biased the results. Finally, this study is only limited to ERI and does not pay attention to more specific clinical problems. It is reported that slight renal injury assessed by cystatin C is also related to cardiovascular disease in patients with chronic kidney disease [19]. Whether qRSN is also related to cardiovascular events will be further discussed in the next study.

In summary, there is an obvious association between qRSN and ERI in patients with arterial hypertension: the risk of ERI rises as qRSN increases. In clinical practice, RSN based on abdominal CT images is easy to obtain and has the potential to indicate the risk of ERI in patients with arterial hypertension from an imaging perspective. Importantly, the results of this study will contribute to the effective clinical prevention and comprehensive management of ERI.

## Figures and Tables

**Table 1:** Clinical characteristics and quantitative classification of renal surface nodularity in patients with arterial hypertension.

Index	Early renal injury group (*n* = 60)	Control group (*n* = 83)	*t*, *Z*, *χ*^2^ values	*P* values
Age	48.100 ± 9.152	48.060 ± 8.069	−0.389	0.697^†^

Sex, male	47 (78.3)	58 (69.9)	1.276	0.259^*∗*^

Hypertension grade			3.603	**<0.001** ^*∗*^
Grade 1	36 (60.0)	61 (73.5)		
Grade 2	8 (13.3)	20 (24.1)		
Grade 3	16 (26.7)	2 (2.4)		

Hypertension course			5.585	**0.061** ^*∗*^
0–9 years	41 (68.3)	66 (79.5)		
10–19 years	9 (15.0)	13 (15.7)		
≥20 years	10 (16.7)	4 (4.8)		

TC (mmol/L)	4.540 ± 1.012	4.480 ± 1.079	−0.314	0.754^ǂ^
TG (mmol/L)	2.031 ± 1.022	2.005 ± 1.745	−1.761	**0.078** ^†^
LDL (mmol/L)	2.654 ± 0.874	2.519 ± 0.816	−0.894	0.373^ǂ^
qRSN	2.080 ± 0.271	1.885 ± 0.270	−3.862	**<0.001** ^†^

Note: all data are the mean ± standard deviation or frequencies (%). *∗χ*^*2*^ test; ^†^ Wilcoxon rank-sum test; ^ǂ^two independent sample *t* tests. LDL, low-density lipoprotein. TC, total cholesterol. TG, triglycerides. qRSN, quantitative classification of renal surface nodularity. A two-sided *P* < 0.1 was considered statistically significant.

**Table 2:** Multiple logistic analysis of early renal injury inpatients with arterial hypertension.

Index	A model without qRSN			A model with qRSN		
	*B*	OR (95% CI)	*P* value	*B*	OR (95% CI)	*P* value
Hypertension grade			0.001			0.005
Grade 1		1			1	
Grade 2	−0.490	0.613 (0.224∼1.674)	0.339	−0.485	0.616 (0.213∼1.778)	0.370
Grade 3	2.652	14.182 (3.016∼66.689)	0.001	2.414	11.183 (2.322∼53.865)	0.003

Hypertension course			0.183			0.474
0–9 years		1			1	
10–19 years	−0.166	0.847 (0.291∼2.466)	0.760	−0.397	0.672 (0.209∼2.159)	0.505
≥20 years	1.207	3.343 (0.872∼12.813)	0.078	0.810	2.248 (0.516∼9.799)	0.281
TG (mmol/L)	−0.044	0.957 (0.732∼1.251)	0.748	−0.068	0.934 (0.700∼1.246)	0.644
qRSN	—	—	—	2.665	14.365 (2.746∼75.146)	0.002

Note: qRSN is the quantitative classification of renal surface nodularity. CI, confidence interval. OR, odds ratio. TG, triglycerides.

## Data Availability

The data supporting this research article are available from the corresponding author on reasonable request.

## Supplementary Materials

Supplementary Materials Table S1: interrater agreement of quantitative classification of renal surface nodularity. Annex 1: clinical data and laboratory results of urea nitrogen and creatinine in patients with arterial hypertension.
