# Supplementary material for: Association between Quantitative Classification of Renal Surface Nodularity and Early Renal Injury in Patients with Arterial Hypertension
Source: Int J Hypertens. 2022 Mar 4;2022:1553700. doi: 10.1155/2022/1553700 (PMC8916879; doi:10.1155/2022/1553700)
Supplement: Supplementary Materials — Table S1: interrater agreement of quantitative classification of renal surface nodularity. Annex 1: clinical data and laboratory results of urea nitrogen and creatinine in patients with arterial hypertension. [file 1553700.f1.zip › 1553700.f1/Annex1.docx]

| name | sex | age | Inpatient number | Image number | creatinine | μmol/L | Cystatin C | mg/L |
| --- | --- | --- | --- | --- | --- | --- | --- | --- |
| chaoyujun | male | 46 | 1138380 | CT-122841 | negative | 81 | negative | 0.78 |
| chenaijuan | female | 52 | 1172955 | CT-128370 | negative | 52 | positive | 1.13 |
| chenfei | female | 43 | 1211400 | C16-518786 | negative | 50 | negative | 0.75 |
| chenhai | male | 47 | 1003034 | DCT-046904 | negative | 84 | negative | 0.68 |
| chenhu | male | 49 | 1308321 | CT-140216 | negative | 66 | positive | 1.23 |
| chenjianguo | male | 59 | 1075423 | C64-094820 | negative | 79 | positive | 1.14 |
| chenshunli | male | 42 | 1000957 | DCT-046122 | negative | 60 | positive | 1.08 |
| chenshuyang | male | 44 | 1234748 | DCT-086066 | negative | 112 | negative | 1.01 |
| chenxifeng | female | 56 | 1191533 | DCT-080187 | negative | 55 | negative | 0.78 |
| chenxu | male | 51 | 1143559 | DCT-073534 | negative | 85 | positive | 1.19 |
| chenyueping | male | 49 | 1108492 | DCT-068218 | negative | 99 | negative | 1.02 |
| chenzhongyang | male | 52 | 1151709 | DCT-075620 | negative | 72 | negative | 0.78 |
| chuwenshu | male | 49 | 1190100 | CT-129671 | negative | 72 | negative | 0.98 |
| daiyingchun | female | 44 | 1043042 | C64-090699 | negative | 96 | positive | 1.23 |
| daizhongxiang | male | 44 | 964683 | DCT-038466 | negative | 75 | negative | 0.96 |
| dansuifu | male | 39 | 1154416 | CT-125590 | negative | 102 | positive | 1.15 |
| dingjumei | female | 56 | 1189061 | DCT-079883 | negative | 73 | negative | 0.75 |
| duchunai | female | 57 | 1197954 | DCT-081500 | negative | 63 | negative | 0.68 |
| duliancai | male | 55 | 1256846 | C16-524096 | negative | 80 | positive | 1.44 |
| fanjunjie | male | 30 | 843469 | C64-059691 | negative | 65 | positive | 1.36 |
| fengmingzhu | female | 56 | 1317056 | REV-015913 | negative | 52 | positive | 1.2 |
| gaofei | male | 42 | 1118779 | C64-100577 | negative | 70 | negative | 0.84 |
| gaojian | male | 41 | 1106500 | DCT-067831 | negative | 65 | negative | 0.86 |
| gaozhiping | male | 44 | 952636 | DCT-036922 | negative | 60 | negative | 0.69 |
| gehongmei | female | 59 | 1257354 | DCT-088697 | negative | 47 | negative | 0.84 |
| gengjidi | female | 50 | 988823 | DCT-044026 | negative | 109 | positive | 1.12 |
| gesuqin | female | 51 | 1286000 | C16-530339 | negative | 63 | positive | 1.18 |
| gukang | male | 57 | 1166898 | DCT-077502 | negative | 97 | positive | 1.26 |
| guwenxing | male | 54 | 976202 | DCT-041734 | negative | 77 | negative | 0.68 |
| guxuehui | female | 53 | 1293343 | CT-139982 | negative | 35 | negative | 0.65 |
| hanyun | female | 60 | 979943 | C64-080543 | negative | 67 | negative | 0.86 |
| hanzhongyin | male | 33 | 1163457 | DCT-077075 | negative | 67 | negative | 0.73 |
| helinhua | male | 51 | 1155234 | DCT-075842 | negative | 96 | positive | 1.78 |
| hewenxin | male | 35 | 1144755 | DCT-073681 | negative | 93 | negative | 0.95 |
| heyeqing | female | 44 | 1243819 | DCT-086907 | negative | 63 | negative | 0.92 |
| heyongqian | male | 57 | 1191017 | DCT-080115 | negative | 66 | positive | 1.09 |
| hongbin | male | 46 | 801307 | DCT-007644 | negative | 58 | negative | 0.88 |
| huangjianrong | male | 58 | 1126386 | C64-101893 | negative | 84 | positive | 1.36 |
| huangshibin | male | 51 | 1014299 | C64-086068 | negative | 51 | negative | 0.78 |
| huaungshenghui | male | 28 | 1170620 | GE64-063541 | negative | 101 | positive | 1.57 |
| hukai | male | 35 | 1150801 | DCT-075571 | negative | 76 | positive | 1.1 |
| huliping | male | 42 | 1172185 | DCT-077973 | negative | 90 | positive | 1.1 |
| huwenyu | male | 32 | 1108486 | DCT-069596 | negative | 68 | positive | 1.16 |
| huzechao | male | 48 | 1008736 | DCT-048788 | negative | 73 | negative | 0.56 |
| jiangahong | female | 58 | 1155794 | ICT-002814 | negative | 59 | positive | 1.09 |
| jiangjianhua | male | 56 | 1100471 | DCT-066712 | negative | 95 | negative | 0.8 |
| jiangjianrong | male | 50 | 1257841 | ICT-020325 | negative | 78 | negative | 0.77 |
| jiangweidong | male | 52 | 1119500 | C64-100661 | negative | 78 | positive | 1.05 |
| jiangxin | male | 27 | 1111499 | DCT-069597 | negative | 84 | negative | 0.69 |
| jiangyuexin | male | 54 | 989056 | DCT-043695 | negative | 75 | negative | 0.84 |
| jiangyuzhong | male | 35 | 1188155 | CT-129401 | negative | 93 | negative | 0.67 |
| jiangzhengyu | male | 47 | 937055 | C64-074410 | negative | 64 | positive | 1.78 |
| jiguowen | male | 50 | 1044666 | DCT-055836 | negative | 74 | positive | 1.36 |
| lifugen | male | 53 | 1009819 | DCT-049041 | negative | 68 | negative | 0.82 |
| limingjun | male | 32 | 1062700 | DCT-058494 | negative | 102 | positive | 1.55 |
| liuchunlin | male | 52 | 1061818 | C64-092715 | negative | 81 | negative | 1.01 |
| liuliangquan | male | 58 | 1080364 | DCT-063195 | negative | 86 | positive | 1.09 |
| liuyoulan | female | 49 | 1205034 | DCT-081800 | negative | 67 | positive | 1.06 |
| liyahong | female | 53 | 1299635 | DCT-093993 | negative | 64 | negative | 0.86 |
| liying | male | 48 | 968516 | DCT-040430 | negative | 68 | negative | 0.69 |
| lizhen | female | 34 | 1311152 | C64-183286 | negative | 43 | negative | 0.85 |
| lucuicui | female | 34 | 1101751 | C64-098565 | negative | 61 | negative | 0.78 |
| luojianchun | male | 55 | 1148187 | DCT-075109 | negative | 81 | negative | 0.61 |
| lvbo | male | 32 | 1206430 | CT-131776 | negative | 104 | positive | 1.36 |
| mazhaoniu | male | 48 | 1255082 | DCT-088437 | negative | 86 | positive | 1.2 |
| miuxiaodan | female | 57 | 1163819 | ICT-004245 | negative | 65 | negative | 0.69 |
| panlanfang | female | 56 | 1291109 | DCT-092957 | negative | 90 | positive | 1.14 |
| pantao | male | 33 | 799275 | C64-051630 | negative | 109 | positive | 1.23 |
| pihaifa | male | 49 | 974044 | DCT-041229 | negative | 66 | negative | 0.84 |
| pujianyuan | male | 59 | 811413 | C64-053546 | negative | 77 | positive | 1.28 |
| qiancaiying | female | 56 | 1047353 | C64-090842 | negative | 63 | positive | 1.27 |
| qiangwusheng | male | 37 | 1008121 | C64-085128 | negative | 78 | negative | 0.82 |
| qianliming | male | 59 | 1000934 | DCT-046644 | negative | 83 | negative | 0.61 |
| qianzhifeng | male | 42 | 1196951 | CT-130317 | negative | 71 | negative | 0.82 |
| shangwenxian | female | 45 | 1255069 | DCT-088436 | negative | 59 | negative | 0.66 |
| shengguoping | female | 59 | 1100369 | C64-098564 | negative | 64 | negative | 0.72 |
| shenweiyuan | female | 49 | 1180492 | C16-515083 | negative | 70 | positive | 1.13 |
| sunjinming | male | 52 | 1285084 | REV-012532 | negative | 88 | positive | 1.22 |
| sunyi | male | 38 | 951446 | C64-076428 | negative | 95 | negative | 0.69 |
| sunzhonglin | male | 36 | 1114444 | DCT-069632 | negative | 64 | negative | 0.85 |
| wangaiqin | female | 56 | 1095729 | C64-097663 | negative | 56 | negative | 0.96 |
| wangaoyun | male | 47 | 887562 | DCT-023169 | negative | 64 | negative | 0.69 |
| wangchangsong | male | 47 | 972083 | DCT-040750 | negative | 75 | negative | 0.66 |
| wangfengying | female | 60 | 1062609 | C64-093498 | negative | 47 | negative | 0.73 |
| wangfulin | male | 59 | 980262 | C64-080685 | negative | 66 | negative | 0.95 |
| wangguiju | female | 40 | 915282 | DCT-028799 | negative | 46 | negative | 0.82 |
| wangguitao | male | 41 | 1112158 | CT-118739 | negative | 66 | positive | 1.21 |
| wanghuachun | male | 46 | 863350 | C64-062653 | negative | 58 | negative | 0.65 |
| wangjianrong | male | 50 | 1150257 | DCT-075135 | negative | 86 | negative | 0.88 |
| wangpeng | male | 33 | 1022479 | C64-087353 | negative | 72 | negative | 0.97 |
| wangyouyuan | male | 56 | 1138672 | DCT-073052 | negative | 120 | negative | 0.55 |
| wangyuancheng | male | 41 | 1246394 | DCT-087143 | negative | 61 | positive | 1.11 |
| wangyuhe | male | 59 | 1303350 | C16-533632 | negative | 81 | positive | 1.32 |
| wangyulun | male | 57 | 1327415 | DCT-098450 | negative | 85 | positive | 1.29 |
| wanyunyu | male | 39 | 1235710 | DCT-085814 | negative | 83 | negative | 0.88 |
| weilixin | male | 49 | 1246957 | DCT-087627 | negative | 98 | negative | 0.69 |
| weiping | female | 50 | 1177059 | DCT-078582 | negative | 78 | negative | 0.65 |
| wuweiguo | male | 48 | 1040019 | DCT-054613 | negative | 101 | negative | 0.75 |
| xiajifa | male | 55 | 915954 | DCT-029621 | negative | 98 | negative | 0.69 |
| xiefei | male | 41 | 1247606 | DCT-087261 | negative | 77 | negative | 0.98 |
| xiejingbo | male | 32 | 1098645 | DCT-067060 | negative | 82 | negative | 0.91 |
| xucuifang | female | 45 | 1226666 | DCT-084366 | negative | 54 | negative | 1 |
| xuewei | male | 49 | 1296589 | REV-013724 | negative | 84 | positive | 1.12 |
| xueyulan | female | 48 | 1106511 | CT-117423 | negative | 40 | negative | 0.61 |
| xujianfang | male | 54 | 914579 | DCT-029428 | negative | 75 | negative | 0.78 |
| xujiang | male | 55 | 996731 | C64-082945 | negative | 56 | negative | 0.78 |
| xujianxin | male | 59 | 1193263 | CT-129863 | negative | 91 | positive | 1.16 |
| xupingjuan | female | 44 | 1199867 | DCT-081368 | negative | 62 | positive | 1.14 |
| yangbin | male | 33 | 1011094 | DCT-049242 | negative | 107 | positive | 1.36 |
| yangluqin | female | 44 | 980597 | C64-080411 | negative | 93 | positive | 1.15 |
| yangzhifang | male | 47 | 1246921 | DCT-087244 | negative | 56 | positive | 1.32 |
| yaoyunfang | female | 48 | 1151726 | DCT-075570 | negative | 69 | negative | 0.69 |
| yinshunlong | male | 56 | 1202473 | DCT-081947 | negative | 80 | positive | 1.47 |
| yinxihua | male | 58 | 1150736 | DCT-075242 | negative | 71 | negative | 0.65 |
| yuanyongfeng | male | 41 | 1019353 | C64-087084 | negative | 82 | negative | 0.81 |
| yunhanrong | male | 55 | 991661 | C64-082059 | negative | 114 | negative | 1 |
| yuwei | male | 49 | 1228714 | CT-133177 | negative | 70 | positive | 1.33 |
| yuxingzhong | male | 54 | 1179117 | DCT-078806 | negative | 90 | positive | 1.42 |
| zangguoqing | male | 53 | 1132614 | C64-102444 | negative | 90 | negative | 0.99 |
| zhaiguoping | male | 58 | 986506 | C64-081276 | negative | 76 | negative | 0.96 |
| zhangcaiqi | male | 56 | 1222341 | CT-132073 | negative | 65 | positive | 1.13 |
| zhangguangshan | male | 38 | 1222377 | DCT-084300 | negative | 99 | positive | 1.05 |
| zhangguangxin | male | 57 | 1150232 | CT-126911 | negative | 36 | negative | 0.83 |
| zhangjie | female | 36 | 1123735 | C64-101170 | negative | 83 | positive | 1.06 |
| zhangminjiang | male | 45 | 1071837 | DCT-059827 | negative | 96 | negative | 0.53 |
| zhangshun | male | 48 | 1230298 | DCT-085255 | negative | 100 | positive | 1.52 |
| zhangyangxian | male | 33 | 1287640 | DCT-092727 | negative | 93 | positive | 1.3 |
| zhangyu | female | 43 | 1035113 | C64-089092 | negative | 70 | negative | 0.84 |
| zhangzaibin | male | 48 | 1221107 | DCT-083915 | negative | 70 | negative | 0.69 |
| zhazhuoying | female | 58 | 1200042 | DCT-081645 | negative | 51 | negative | 0.58 |
| zhongshengwei | male | 52 | 1103619 | C64-098724 | negative | 99 | negative | 0.64 |
| zhoujunqiang | male | 48 | 985065 | DCT-040382 | negative | 109 | negative | 0.99 |
| zhoumei | female | 50 | 1287788 | REV-013042 | negative | 60 | negative | 0.84 |
| zhousongzhao | male | 59 | 1166767 | DCT-077707 | negative | 88 | positive | 1.22 |
| zhoutingyou | male | 54 | 1134120 | DCT-072473 | negative | 76 | negative | 0.88 |
| zhouyuanjuan | female | 56 | 1044678 | PC16-090356 | negative | 68 | negative | 0.92 |
| zhuhuosheng | male | 57 | 1163199 | CT-126407 | negative | 88 | positive | 1.3 |
| zhujinfang | male | 57 | 946738 | DCT-035361 | negative | 99 | positive | 1.72 |
| zhukuan | male | 29 | 986486 | C64-081303 | negative | 71 | negative | 0.61 |
| zhuruping | male | 56 | 1211784 | DCT-082912 | negative | 77 | positive | 1.32 |
| zhutao | male | 40 | 1326520 | DCT-098513 | negative | 86 | positive | 1.28 |
| zhuzhengyi | male | 45 | 1262113 | ICT-022947 | negative | 73 | positive | 1.2 |
| zuomingbao | male | 59 | 1189153 | DCT-079985 | negative | 88 | positive | 1.32 |

| Normal Cystatin C standard was followed: | | | |
| --- | --- | --- | --- |
| cystatin C ≤ 1.02 mg/L. | | |  |
|  |  |  |  |
| Normal serum creatinine standard was followed: | | |  |
| •0-16 years old: 35-105 μmol/L ; | | |  |
| •Man: 17-49 years old, 48-110 μmol/L ; ≥ 50 years old, 48-127 μmol/L | | | |
| •Woman: ≥ 17 years old, 48-110 μmol/L | | | |
