# Supplementary material for: Association between Quantitative Classification of Renal Surface Nodularity and Early Renal Injury in Patients with Arterial Hypertension
Source: Int J Hypertens. 2022 Mar 4;2022:1553700. doi: 10.1155/2022/1553700 (PMC8916879; doi:10.1155/2022/1553700)
Supplement: Supplementary Materials — Table S1: interrater agreement of quantitative classification of renal surface nodularity. Annex 1: clinical data and laboratory results of urea nitrogen and creatinine in patients with arterial hypertension. [file 1553700.f1.zip › 1553700.f1/tableS1.docx]

**Table S1** Interrater agreement of quantitative classification of renal surface nodularity

| No. | Observer 1 | Observer 2 | Group |
| --- | --- | --- | --- |
| 1 | 1.977 | 1.886 | renal injury group |
| 2 | 1.974 | 1.974 | renal injury group |
| 3 | 1.894 | 1.894 | renal injury group |
| 4 | 2.367 | 2.436 | renal injury group |
| 5 | 2.189 | 2.262 | renal injury group |
| 6 | 1.731 | 1.803 | renal injury group |
| 7 | 1.970 | 1.921 | renal injury group |
| 8 | 2.110 | 2.162 | renal injury group |
| 9 | 2.063 | 2.063 | control group |
| 10 | 1.610 | 1.610 | control group |
| 11 | 2.155 | 1.994 | control group |
| 12 | 1.526 | 1.526 | control group |
| 13 | 1.705 | 1.705 | control group |
| 14 | 2.076 | 2.127 | control group |
| 15 | 2.345 | 2.342 | control group |
| 16 | 1.747 | 1.811 | control group |
| 17 | 1.788 | 1.773 | control group |
| 18 | 1.921 | 1.921 | control group |
| 19 | 1.428 | 1.442 | control group |
| 20 | 2.237 | 2.289 | control group |
|  | 1.941±0.263* | 1.947±0.270* |  |

Note: * Means ± standard deviation. There was no significant difference between the two groups (*t*=-0.076, *P*=0.940).
